# Effectiveness of Tailor-Made Physiotherapy Protocol in Smartphone-Addicted Individuals With Text Neck Syndrome and Short Message Service (SMS) Thumb

**DOI:** 10.7759/cureus.57453

**Published:** 2024-04-02

**Authors:** Harsh R Nathani, Pratik Phansopkar

**Affiliations:** 1 Musculoskeletal Physiotherapy, Ravi Nair Physiotherapy College, Datta Meghe Institute of Higher Education and Research, Wardha, IND

**Keywords:** personalized treatment, pain management, smartphone addiction, musculoskeletal disorders, physiotherapy, sms thumb, text neck syndrome, smartphone usage

## Abstract

Background

Smartphone usage has led to an increase in text neck syndrome (TNS) and short message service (SMS) thumb, causing neck, shoulder, and thumb pain, affecting daily activities. Limited treatment options are available for these conditions, and early intervention is crucial to prevent chronic pain and musculoskeletal issues. This study sought to determine the impact of a personalized physiotherapy treatment plan on alleviating TNS and SMS thumb in individuals who demonstrate excessive smartphone usage.

Method

The study utilized a single-group, pre- and post-study design with a sample size of 54 smartphone-addicted individuals. The intervention involved a personalized physiotherapy protocol for three weeks, and data was collected using a visual analog scale (VAS), Neck Disability Index (NDI), Smartphone Addiction Scale (SAS), Cornell Hand Discomfort Questionnaire (CHDQ), and range of motion (ROM).

Results

Descriptive statistics showed a significant reduction in mean scores from pre- to post-rehabilitation, indicating the potential effectiveness of the treatment. Hypothesis testing revealed significant improvements in VAS, NDI, and CHDQ scores post-rehabilitation, with a p-value of <0.05. Correlation analysis demonstrated moderate to strong correlations between pre- and post-rehabilitation scores for VAS, NDI, and CHDQ, with age showing minimal influence on treatment outcomes.

Conclusion

The findings emphasize the efficacy of personalized interventions in addressing smartphone-related musculoskeletal disorders and underscore the need for further research to optimize treatment protocols and long-term outcomes.

## Introduction

As a prevalent ailment, neck discomfort has become a common and extensive cause of impairment worldwide. Interestingly, the incidence of neck pain is still relatively high, regardless of age, similar to the prevalence of low back pain [1]. An astounding 73% of college students and 64.7% of people working remotely report having neck or back discomfort, according to epidemiological data [2]. The term “posture” describes how the human body is anatomically arranged to withstand the pull of gravity while sitting, standing, or moving. Different forms of postural distortion are examined and identified using the posture quadrants categorization system. One particular form of postural distortion that mostly affects the head and neck is called frontal or forward head posture (FHP). Improper neck posture during activities such as texting on cell phones and reading on personal computers can give rise to a complex cluster of clinical symptoms known as “text neck syndrome” (TNS). Patients with a high risk of developing TNS are identified and screened early using posture imaging methods, clinical examinations, and medical histories [3].

This syndrome is further exacerbated by improper sleeping postures and FHP, which can lead to cervical spatial changes, respiratory dysfunction, reduced range of motion (ROM), temporomandibular joint issues, diminished vital capacity, impaired proprioception, and carpal tunnel syndrome [4]. Higher flexion degrees apply additional stress to the spine, increasing the weight-bearing load and producing a weight increase from 18.14 kg at 30° to 27.22 kg at 60° [5]. The cervical spine is elevated in cases of FHP, causing changes in cervical muscles and disrupting balance mechanisms [6]. This leads to a shift in the center of gravity (COG), affecting the entire body’s COG and postural control. FHP also affects thoracic wall muscles, cervical spine, head muscles, supra- and infrahyoid muscles, middle trapezius, and rhomboids [7]. Chronic FHP can cause sternocleidomastoid muscles to shorten, tighten, and weaken, affecting the hyoid bone. FHP can affect cervical sensorimotor control and respiratory function and is associated with reduced proprioception [8]. A two-week home exercise regimen for TNS improves postural alignment and alleviates discomfort. Other interventions include posture training, oculomotor exercises, breathing exercises, and ergonomic modifications. Proper posture improves ventilation, benefiting the body and nervous system [9].

The technology column discusses a new ailment that causes distressing musculoskeletal symptoms called texting thumb, sometimes known as texting tendinitis. The fact that the typical person texts for 55 minutes per day shows how much of an influence this disease has on people [10]. Thumb texting is frequently associated with De Quervain’s tenosynovitis, which is defined as inflammation of the wrist tendons near the base of the thumb; it is sometimes called as blackberry thumb or washerwoman’s sprain [11]. The Finkelstein test diagnoses De Quervain’s tenosynovitis by testing the patient’s extensor tendons, indicating discomfort during activities [12]. Research on the correlation between musculoskeletal disorders and smartphone usage has revealed a substantial effect on students pursuing physical therapy [13]. Depending on hand size, there may be variations in the risk of musculoskeletal diseases when using smartphones, as shown by variations in thumb motions and activity [14]. Thumb surgery like tenosynovectomy can precisely restore some of the functioning, but undergoing physiotherapy rehabilitation is the most recommended approach. One way to lessen these problems is to text both people at the same time, take regular breaks, avoid typing quickly, and undergo exercises [15]. The objective of our study was to evaluate the effectiveness of a customized physiotherapy protocol on reducing pain and discomfort associated with TNS and short message service (SMS) thumb in smartphone-addicted individuals.

## Materials and methods

This research was conducted at Acharya Vinoba Bhave Rural Hospital (AVBRH), Wardha, India, after receiving approval from the Institutional Ethics Committee of Datta Meghe Institute of Higher Education and Research, deemed to be University. The study focused on patients experiencing symptoms of TNS and SMS thumb. It was carried out over a period of six months, from August 2023 to February 2024, with each patient undergoing a three-week rehabilitation program. The study received approval from the institutional ethics committee under the IEC approval number: DMIHER(DU)/IEC/2023/1002.

Procedure

An informed consent was taken from participants aged 18-30 years, with smartphone addiction that was evaluated using the Smartphone Addiction Scale, TNS, SMS thumb, and those who had English language proficiency. The outcome measures were recorded on days 1 and 21, and data was analyzed. The baseline outcome measures of visual analog scale (VAS), Neck disability index (NDI), and Cornell Hand Discomfort Questionnaire (CHDQ) were assessed pre-intervention day 1 and after three weeks post-intervention day 21.

For text neck syndrome

**Figure 1:**
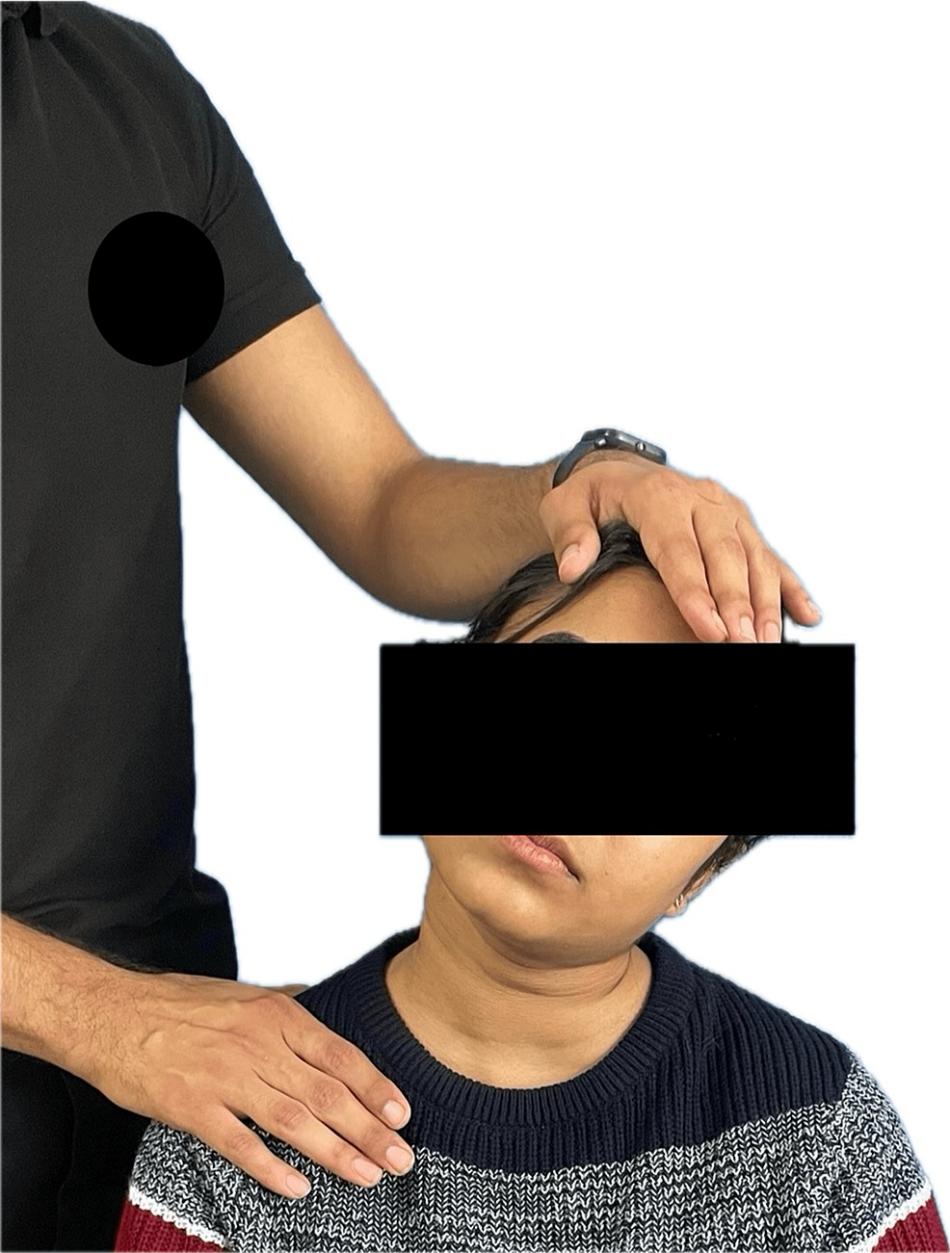
Manual stretching for trapezius muscle to reduce neck discomfort caused by text neck syndrome

Perform cervical ROM exercises, such as flexion, extension, rotations, and lateral flexion, 10 times, three times a day. Maintain smooth, controlled movements. Perform trapezius stretching by tilting your head toward one shoulder, and the therapist will apply a stretch and hold for 10-30 seconds, as shown in Figure 1. Perform chin and scapular retractions by tucking your chin toward your chest and holding for 10 seconds. Repeat each exercise for 10 repetitions. Allow adequate rest between sessions, avoid prolonged smartphone use, use ergonomic accessories or posture correction techniques, and consider periodic breaks from smartphone usage to allow neck muscles to recover.

For SMS thumb

**Figure 2:**
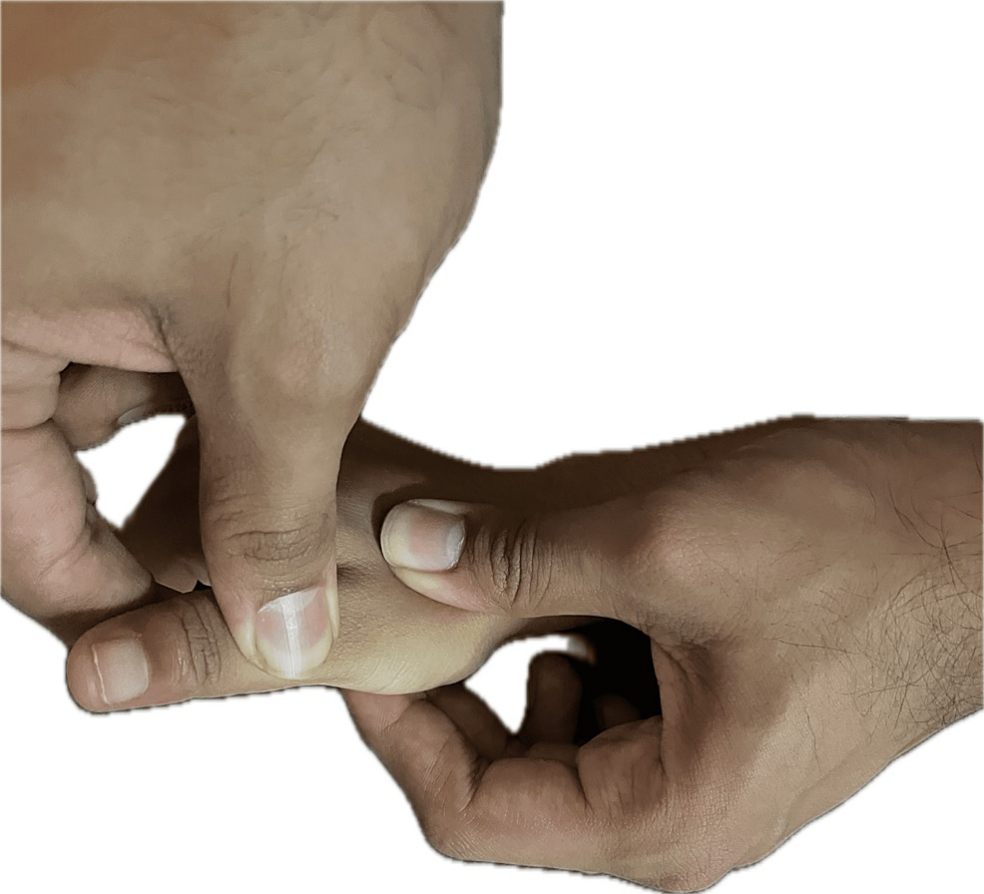
Mobilization technique for metacarpophalangeal joint of thumb for treating SMS thumb SMS, short message service

Apply ultrasound at a frequency of 3 MHz with an intensity of 0.6 W/cm for three minutes. Ensure proper coupling gel application and use appropriate ultrasound technique to target the affected thumb region. Maintain consistent movement of the ultrasound transducer to evenly distribute thermal energy and promote tissue healing. Perform gentle mobilization techniques to improve joint flexibility and reduce stiffness in the metacarpophalangeal joint. Use graded oscillatory movements or sustained stretches under the guidance of a trained therapist, as shown in Figure 2. Avoid excessive force or aggressive manipulation to prevent exacerbation of symptoms. Strengthening exercises focus on the extensor pollicis brevis and abductor pollicis longus muscles to support thumb function, which is done by squeezing the gel ball.

Statistical analysis

The statistical analysis was carried out using the paired sample approach to test our hypothesis. This method was chosen as it enabled us to compare the same group of individuals before and after the intervention. The analysis included the utilization of various statistical tests, such as Fisher’s exact test and student’s paired and unpaired t-tests. We employed IBM SPSS Statistics, version 27.0 (IBM Corp., Armonk, NY) and GraphPad Prism, version 7.0, as the software for analysis, with a significance level of p < 0.05 being deemed as the level of significance.

## Results

**Figure 3:**
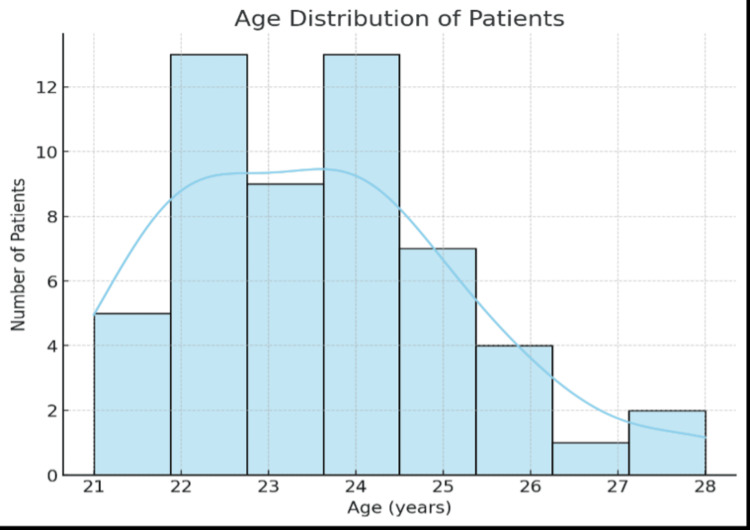
Graphical representation of the distribution of patients according to age

A total of 54 patients were included in the study. All the 54 patients were taken in one single group. All of them were given individualized physiotherapy protocol for TNS and SMS thumb. They were distributed according to their age, as shown in Figure 3.

**Figure 4:**
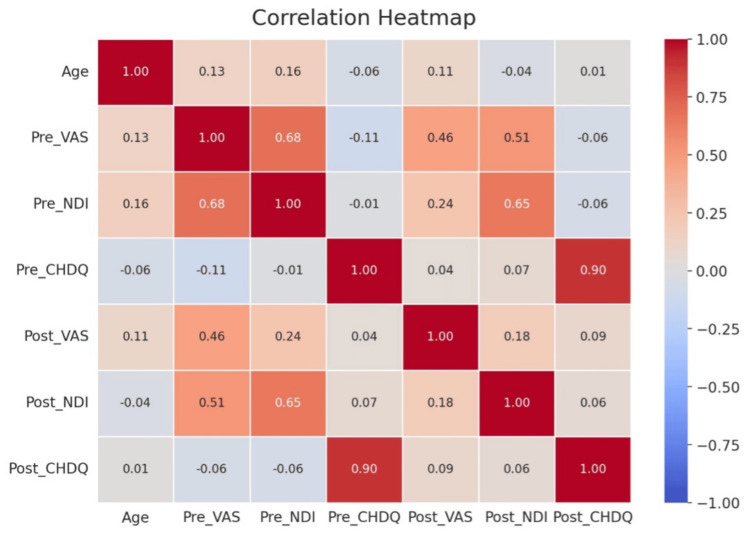
Heatmap correlation of VAS, NDS, and CHDQ with its pre- and post-intervention VAS, visual analog scale; NDI, Neck Disability Index; CHDQ, Cornell Hand Discomfort Questionnaire

A statistically significant difference was found in VAS, NDI, and CHDQ in all 54 patients, which is represented in the correlation heatmap, as shown in Figure 4. The statistical difference is shown in Table 1, which illustrates the effectiveness of tailor-made physiotherapy protocol for SMS thumb and TNS after three weeks of treatment.

**Table 1:** Results of paired sample t-test comparing pre- and post-rehabilitation outcomes VAS, visual analog scale; NDI, Neck Disability Index; CHDQ, Cornell Hand Discomfort Questionnaire

Variable outcome	T-statistic	p-value
VAS	19.83	<0.05 (3.50e-26)
NDI	17.85	<0.05 (4.50e-24)
CHDQ	7.15	<0.05 (2.59e-09)

The results highlighted that tailor-made physiotherapy protocol when used in patients with SMS thumb and TNS proved that the customized physiotherapy protocol is effective in managing SMS thumb and TNS, resulting in alleviation of pain (VAS), improved NDI, and reduced hand discomfort (CHDQ).

## Discussion

This study aims to evaluate the effectiveness of a personalized physiotherapy treatment plan in managing TNS and SMS thumb in smartphone-addicted individuals. The objectives include assessing the pain reduction and functionality of the intervention, as well as assessing the prevalence and severity of these conditions among smartphone-addicted individuals. A sample size of n = 54 was evaluated, and a three-week intervention was used to tailor the management for TNS and SMS thumb, which resulted in significant improvements in outcomes such as VAS, NDI, and CHDQ. Descriptive statistics revealed a predominantly female cohort in their early twenties, with approximately 85.19% females and 14.81% males. The mean pre-rehabilitation VAS score was 6.61, decreasing significantly to 2.89 post-rehabilitation. Similarly, the NDI decreased from 27% to 15% and the CHDQ from 6.74 to 3.31. Hypothesis testing confirmed significant improvements in VAS, NDI, and CHDQ scores post-rehabilitation, with t-statistics of 19.83, 17.85, and 7.15, respectively, all yielding p-values <0.05. Correlation analysis demonstrated moderate to strong correlations between pre- and post-rehabilitation scores for VAS, NDI, and CHDQ, with age showing minimal influence on treatment outcomes. Overall, the findings emphasize the efficacy of personalized interventions in addressing smartphone-related musculoskeletal disorders. In Switzerland, 16.9% of teenagers report having a smartphone addiction, with 76% of participants indicating a moderate to high risk.

Long work hours were cited by 27.1% of respondents in a German survey as the reason they were smartphone addicts [16]. Hajihosseini et al. showed that the excessive use of mobile phones, handheld electronic devices with small screens, is a significant contributor to musculoskeletal problems in the head and neck region, as the forward head position places excessive strain on the muscles and cervical joints located at the back of the cervical region [17]. Letafatkar et al., in a study, showed that the deep neck flexors (longus capitis, longus colli, and rectus capitis anterior) experience decreased activation and weakened strength during neck pain, while the superficial muscles (sternocleidomastoid and anterior scalene) fatigue faster and suffer from impaired neuromuscular function. Addressing these concerns can be accomplished through the implementation of a fundamental exercise regimen [18]. Sarraf et al. proved that strengthening and extending the neck’s deep flexors through neck corrective exercises can help reduce the severity of neck pain and NDI while also strengthening shoulder girdle muscles, such as the rhomboid, middle, and lower trapezius, and stretching the pectoralis minor, dorsal, and rotator cuff shoulder can help maintain appropriate head and neck postures [19].

Thumb involvement is common in smartphone use, and numerous studies have explored the connection between De Quervain’s tenosynovitis and different technological devices [12]. SMS thumb results in wrist discomfort in the region of the radial styloid process. Excessive use of the thumb for texting on mobile devices has been linked to De Quervain’s tenosynovitis. The predominant outcome is a positive Finkelstein test. In a research conducted in Pakistan, a high frequency of texting on phones and smartphones was indicated by the positive results of the Finkelstein test in 149 of 300 participants [13]. A similar study in India found that 40% of subjects tested positive for the Finkelstein test [14]. A 2006 study in France revealed that the occurrence rate of De Quervain’s tenosynovitis stands at 1.2%, with males and females having incidences of 0.6% and 2.1%, respectively [20]. The study’s scope was limited due to its short trial duration and lack of long-term follow-up with participants. This may have limited data collection and analysis, potentially limiting the findings and the ability to assess the intervention’s sustained effectiveness over an extended period.

## Conclusions

The study demonstrates that a personalized physiotherapy treatment plan is highly effective in managing TNS and SMS thumb among individuals addicted to smartphone use. Through a meticulous examination of pain levels, neck disability, hand discomfort, and overall well-being, our findings underscore the significant improvements achieved through tailored interventions. The statistically significant reductions in the VAS, NDI, and CHDQ scores post-rehabilitation reaffirm the efficacy of the intervention. These results not only emphasize the importance of addressing musculoskeletal disorders associated with modern technology but also highlight the value of personalized physiotherapy interventions in improving the quality of life for affected individuals. Further research is warranted to optimize treatment protocols and ensure long-term management of these prevalent conditions in smartphone-addicted populations.
